# Fluoreszenzbasierte Konfokalmikroskopie – vollständige Digitalisierung der Pathologie

**DOI:** 10.1007/s00292-024-01311-y

**Published:** 2024-03-06

**Authors:** Andreas G. Loth, Anne Fassl, Felix K. H. Chun, Jens Köllermann, Sylvia Hartmann, Steffen Gretser, Paul K. Ziegler, Nadine Flinner, Falko Schulze, Peter J. Wild, Maximilian N. Kinzler

**Affiliations:** 1 Universitätsklinikum Frankfurt, Klinik für Hals‑, Nasen- und Ohrenheilkunde, Goethe-Universität Frankfurt, Frankfurt am Main, Deutschland; 2 Universitätsklinikum Frankfurt, Klinik für Urologie, Goethe-Universität Frankfurt, Frankfurt am Main, Deutschland; 3 Universitätsklinikum Frankfurt, Dr. Senckenbergisches Institut für Pathologie, Goethe-Universität Frankfurt, Frankfurt am Main, Deutschland; 4 Frankfurt Institute for Advanced Studies (FIAS), Frankfurt am Main, Deutschland; 5 Frankfurt Cancer Institute (FCI), Frankfurt am Main, Deutschland; 6 Universitätsklinikum Frankfurt, Medizinische Klinik 1, Goethe-Universität Frankfurt, Frankfurt am Main, Deutschland

**Keywords:** Digitale Pathologie, Digitaler HE-Schnitt, Schnellschnittdiagnostik, Lebertransplantation, Transplantationspathologie, Digital pathology, Digital HE scan, Frozen section, Liver transplantation, Transplantation pathology

## Abstract

**Hintergrund:**

Mit Hilfe der fluoreszenzbasierten Konfokalmikroskopie (FCM) lassen sich virtuelle HE-Schnitte in Echtzeit erstellen. Bislang findet die FCM Anwendung in der Derma‑/Uro- und Gynäkopathologie. Die FCM eröffnet die Perspektive eines digitalen Gefrierschnitts, der den herkömmlichen Gefrierschnitt in Zukunft ersetzen könnte.

**Ziel der Arbeit (Fragestellung):**

Ziel unserer aktuellen Arbeit ist die Implementierung der FCM als Bestandteil vollständig digitalisierter Abläufe im pathologischen Workflow. Hierfür wird der aktuelle Einsatz der FCM in der Transplantationspathologie auf weitere Fachdisziplinen wie Urologie und HNO ausgeweitet.

**Material und Methoden:**

Der Einsatz der FCM-Technik erfolgt aktuell weiterhin prospektiv bei nativen Gewebeproben potenzieller Spenderlebern. Die herkömmliche Schnellschnittdiagnostik in Gefriertechnik wird vergleichend zu virtuellen FCM-Scans angewandt.

**Ergebnisse:**

Die Daten zeigen eine nahezu perfekte Übereinstimmung für den Nachweis von Cholangitis, Fibrose und Malignität sowie ein hohes Maß an Übereinstimmung für z. B. makrovesikuläre Steatose, Entzündung, Steatohepatitis und Nekrose zwischen virtuellem FCM-Scan und herkömmlichen Schnellschnitt.

**Schlussfolgerung:**

Da die Verfügbarkeit der zeit-, und kostenintensiven Schnellschnittdiagnostik im Rahmen der Transplantationspathologie im Dauerbetrieb (24/7) aufgrund eines zunehmenden Fachkräftemangels mittlerweile nur noch an sehr wenigen universitären Zentren in Deutschland etabliert ist, könnte der Einsatz der FCM-Technik ein wichtiger Baustein im aktuellen Prozess hin zu einer vollständig digitalisierten Pathologie sein und sollte somit auf verschiedene Fachdisziplinen ausgeweitet werden.

## Abstract (en)

**Background:**

Fluorescence-based confocal microscopy (FCM) can be used to create virtual H&E sections in real time. So far, FCM has been used in dermato-, uro-, and gynecopathology. FCM allows the creation of a completely digitized frozen section, which could potentially replace conventional frozen sections in the future.

**Objective:**

The aim of the current work is to implement FCM technology as a component of fully digitized processes in the pathological workflow. For this purpose, the current use of FCM in liver transplant pathology will be extended to other disciplines such as urology and otorhinolaryngology.

**Materials and methods:**

The FCM technique continues to be used prospectively on native tissue samples from potential donor livers. Conventional frozen sections are used comparatively to virtual FCM scans.

**Results:**

The data show a nearly perfect agreement for the detection of cholangitis, fibrosis, and malignancy, and a high level of agreement for, e.g., macrovesicular steatosis, inflammation, steatohepatitis, and necrosis between virtual FCM scans and conventional routine diagnostic frozen sections.

**Conclusion:**

Since the availability of time- and cost-intensive frozen section diagnostics in the context of transplant pathology in continuous operation (24/7) is now only established at very few university centers in Germany due to an increasing shortage of specialists, the use of FCM could be an important building block in the current process leading towards a fully digitized pathology workflow and should thus be extended to various disciplines.

## Hintergrund und Fragestellung

### Hinführung zum Thema

Die FCM ist eine neuartige Technik, bei der sich virtuelle HE-Schnitte von nativem Gewebe in Echtzeit erstellen lassen. Da das native Gewebe nicht fixiert wird, bleibt die nachgeschaltete Diagnostik inklusive immunhistochemischer oder molekularpathologischer Untersuchungen unbeeinträchtigt. Auch die Ex-vivo-Generierung von lebenden Zellmodellen ist möglich. Das Anwendungsspektrum der FCM hat sich in den vergangenen Jahren über den Einsatz in der Dermatologie bis hin zur Transplantationspathologie deutlich erweitert. Insbesondere als Alternative zur herkömmlichen Schnellschnittdiagnostik kann die FCM-Technik zukünftig einen wichtigen Pfeiler zu einer vollständig digitalisierten Pathologie darstellen.

### FCM – Anwendung in der Vergangenheit

In der Vergangenheit hat sich die FCM als Alternative in der Routinediagnostik verschiedener Fachdisziplinen etabliert. So findet die FCM bei dermatologischen Untersuchungen zur Differenzierung gutartiger und bösartiger Hautläsionen [1] oder zur Tumordiagnose von Biopsien der Mamma und der Nieren Anwendung [2, 3]. Weiterhin könnte die FCM-Technik im Vergleich zur konventionellen Histopathologie eine zuverlässige Methode zur Beurteilung von Prostatakrebs darstellen [4–6]. Der Einsatz der FCM-Technik insbesondere in der Leber- und Transplantationspathologie ist bislang jedoch noch unzureichend erforscht. Eine Studie aus dem Jahr 2022 zeigte erstmals, dass die FCM auch die histologische Untersuchung nativer Leberproben ermöglicht [7]. Die diagnostische Aussagekraft war ähnlich wie bei Gefrierschnitten und erlaubte zuverlässige Tumordiagnosen und Aussagen über das Ausmaß entzündlicher Infiltrate oder struktureller Veränderungen, insbesondere makrovesikulärer Steatose [7].

## Studiendesign und Untersuchungsmethoden

### FCM – Vorbereitungen

**Figure 1:**
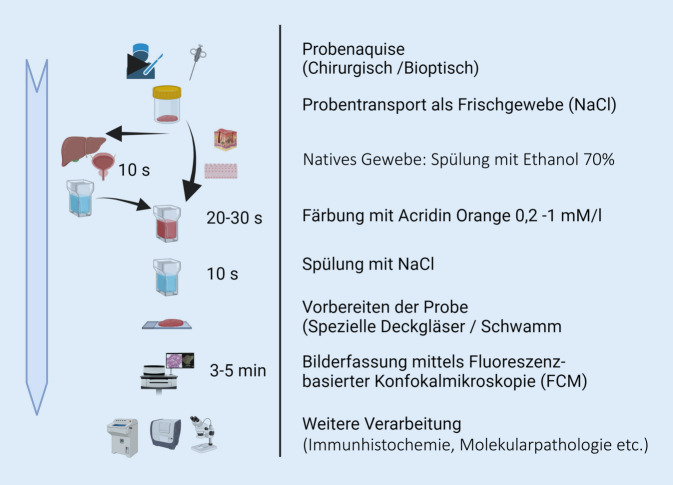

Nach einer Vorbehandlung des nativen Gewebes mit Ethanol (70 %) über 10 s folgt eine Inkubation mit dem Fluoreszenzfarbstoff Acridinorange (AO; 0,6 mM; Sigma-Aldrich, St. Louis, MO, USA) über 30 s. Zwischen den jeweiligen Schritten wird das native Gewebe in physiologischer Kochsalzlösung (0,9 % NaCl) gewaschen. In Abhängigkeit der Probengröße werden ca. 1–2 ml der oben genannten Substanzen benötigt. Anschließend wird die vorbehandelte Gewebeprobe mit einem dünnen Schwamm fixiert, zwischen zwei magnetische Glasträger eingespannt und zur Bildaufnahme ins Mikroskop gelegt. Schwammmaterial sowie Glasträger sind wiederverwendbar. Eine eintägige Vor-Ort-Schulung durch den Hersteller umfasst die Vorbehandlung, die Einstellung der Laser sowie die Funktion des Bildexports. Am Ende erfolgt die Fixierung der Gewebeprobe in 4 % PBS-gepuffertem Formaldehyd. Die Darstellung des Workflows zeigt Abb. 1. Für diese Studie wurde die gesamte FCM im Dr. Senckenbergischen Institut für Pathologie (SIP), Universitätsklinikum Frankfurt, durchgeführt.

### FCM – Funktionsweise und Bilderstellung

Bei der FCM werden digitale Bilder in Echtzeit erstellt, welche den herkömmlichen HE-Schnellschnitten sehr ähnlich sind. Hierfür wird ein Laserstrahl auf einen einzigen Punkt in der Gewebeprobe fokussiert, während die Probe sich reihenweise unterhalb des Lasers verschiebt. Dadurch wird eine Reihe von zweidimensionalen Bildern erzeugt, die dann zu einem dreidimensionalen Bild der Probe zusammengesetzt werden [8]. Das in dieser Studie verwendete Gerät „VivaScope 2500M-G4“ (MAVIG GmbH, VivaScope Systems, München, Deutschland) bietet eine maximale Untersuchungstiefe von 200 μm, eine vertikale optische Auflösung von < 5 μm und eine 550-fache Vergrößerung [9]. Es besteht aus zwei Lasern mit unterschiedlichen Wellenlängen zur Erzeugung von Fluoreszenz- (488 nm) und Reflexionssignalen (638 nm), die gleichzeitig zelluläre (z. B. Zellkerne) sowie zytoplasmatische und extrazelluläre Strukturen der Probe sichtbar machen. Das Fluoreszenzsignal basiert auf dem zuvor applizierten Fluoreszenzfarbstoff Acridinorange. Die Einstellungen können vom Untersucher für jede Probe individuell angepasst werden, basierend auf der Eindringtiefe in das Gewebe, der Gewichtung der beiden Wellenlängen sowie der Inkubationszeit mit Acridinorange vor der Bildgebung. Ein integrierter Algorithmus wandelt die Signale dann in digitale Bilder um. Im Online-Portal ist schließlich eine sofortige Auswertung der Bilder rund um die Uhr, unabhängig vom Standort des FCM-Geräts und ohne die Hilfe eines geschulten Technikers, möglich.

## Ergebnisse

### FCM – aktuelle Anwendung im SIP

**Figure 2:**
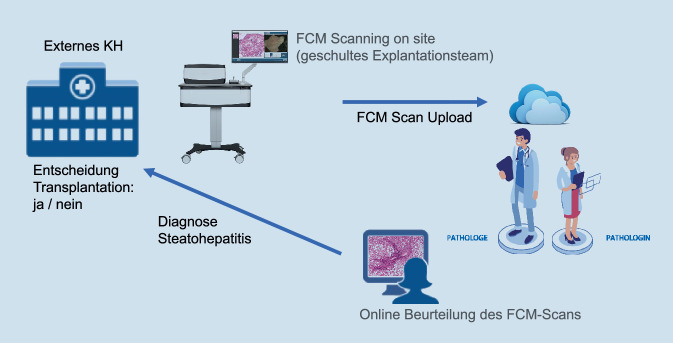

**Figure 3:**
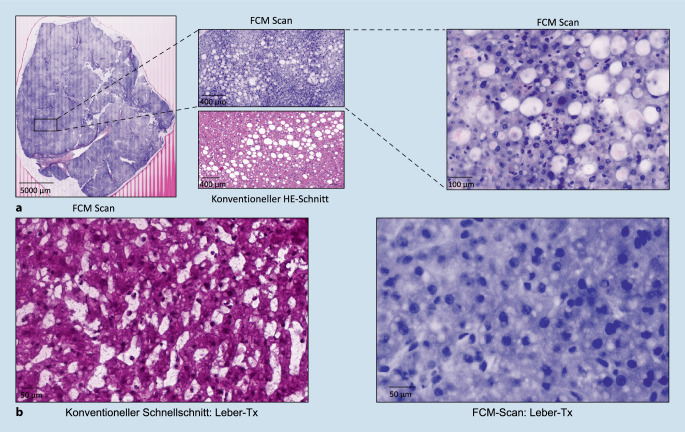

In der Transplantationspathologie ist die FCM noch keine gängige Praxis. Die Verfügbarkeit der Schnellschnittdiagnostik im Dauerbetrieb (24/7) ist mittlerweile jedoch nur noch an sehr wenigen universitären Zentren in Deutschland etabliert, da die Unterhaltung von transplantationspathologischen Instituten in der Regel zeit-, kosten- und personalintensiv ist. Insbesondere der zunehmende Fachkräftemangel und die damit verbundene Schwierigkeit, den Bereitschaftsdienst zur Schnellschnittdiagnostik mit qualifiziertem Laborpersonal nachts, am Wochenende sowie an Feiertagen abzudecken, stellen Universitätsklinika vor große Herausforderungen. Um der in den letzten Jahren stagnierenden Zahl von Lebertransplantationen in Deutschland entgegenzuwirken [10], ist die Identifizierung neuartiger Technologien, die die histopathologische Beurteilung von Spenderpräparaten, die für eine Transplantation in Frage kommen, erleichtern, von größter Bedeutung. Im interdisziplinären Prozess einer Organtransplantation ist insbesondere das Timing entscheidend, da die Organe so schnell wie möglich entnommen werden müssen, um das bestmögliche Ergebnis für den Empfänger und den Erfolg der Transplantation zu gewährleisten. Das Fehlen standardisierter Kriterien für die Beurteilung von Spenderbiopsien und der Mangel an verfügbarem Fachwissen steigern das Dilemma. Die Beurteilung der Spenderproben durch den Pathologen ist oft ein kritischer Punkt im Prozess [11]. Die histologische Bestätigung ist nach wie vor der Referenzstandard für die Beurteilung von Spenderbiopsien, wobei die Steatose der häufigste zu untersuchende Befund ist [12, 13]. Seltener, aber mit sicherer Verwerfung der Spenderorgans verbunden, ist der Nachweis von Malignität [14]. Um den zukünftigen Einsatz der FCM-Technik in der Evaluation von Spenderorganen zu testen, wurden diese sowie Leberbiopsien mittels FCM als auch im konventionellen Schnellschnitt vergleichend untersucht. Die Machbarkeit eines vollständig digitalisierten pathologischen Workflows wurde insgesamt an 20 Leberproben (13 Keilbiopsien im Rahmen von Routineoperationen, 7 Spenderlebern) untersucht. Grundlage der Evaluation waren die von der Deutschen Stiftung Organtransplantation (DSO) gültigen Kriterien zur Begutachtung von Spenderlebern (makro-/mikrovesikuläre Steatose, Steatohepatitis, Fibrose, Entzündung, Nekrose, Cholangitis, Cholestase, Malignität). Die Evaluation erfolgte im Zufallsprinzip durch einen Pathologen mit hepatobiliärem Schwerpunkt. Die histopathologische Bewertung der FCM-Bilder erfolgte „remote“ im Cloud-basierten Online-Portal, in welches die digitalen Scans vom Gerät aus hochgeladen werden können. Die zeitlichen Einsparungen bei der FCM im Vergleich zum konventionellen Schnellschnitt liegen in unserer Studie nur im Bereich weniger Minuten (Tab. 1). Da unsere Studie als „proof of concept“ für einen vollständig digitalisierten pathologischen Workflow mit der Idee des „on-site“ FCM im OP-Saal dienen soll, sind die zukünftigen Einsparpotenziale im Bereich Zeit/Personal weitreichender: der Wegfall des Probentransports vom externen Krankenhaus ins pathologische Institut sowie der Wegfall des MTA-Bereitschaftsdienstes im 24/7-Betrieb (Tab. 2; Abb. 2). Diese beiden Faktoren können entscheidende Stellschrauben in der Aufrechterhaltung einer universitären Transplantationspathologie sein. Die FCM-Bilder werden nach erfolgter Evaluation zusätzlich auf die internen Server des Instituts für Pathologie exportiert und aufbewahrt. Die Arbeit am SIP hob die Stärke der Übereinstimmung zwischen Schnellschnitt und FCM hervor, die dem Vergleich von konventioneller Hämatoxylin-Eosin- und FCM-Bildgebung überlegen war [15]. Insbesondere im Hinblick auf die histopathologische Begutachtung von Transplantatlebern zeigten die Daten eine nahezu perfekte Übereinstimmung für die DSO-Kriterien Cholangitis, Fibrose und Malignität sowie ein hohes Maß an Übereinstimmung für makrovesikuläre Steatose, Entzündung, Steatohepatitis und Nekrose [15]. Vergleichende Bilder von Leberproben in Schnellschnitttechnik sowie mittels FCM sind in Abb. 3 dargestellt. Die Auswertung von Schnellschnitten in Gefrierschnitttechnik kann aufgrund von Artefakten des gefrorenen Lebergewebes sehr variabel sein, was zu Schwankungen bei der Beurteilung potenzieller Transplantate führt [16]. Auch bei der FCM-Bildgebung entstehen streifenförmige Scan-Artefakte. Interessanterweise verschwinden die Scan-Artefakte der digitalen FCM-Scans jedoch im Prozess des Zoomens im Gegensatz zu den Artefakten im Gefrierschnitt. Die FCM kann somit einen Wendepunkt einer digitalen Transplantationspathologie darstellen, da es sich um eine materialschonende Methode handelt, die eine schnelle diagnostische Rückmeldung ermöglicht. Dies kann zu schnelleren und sichereren therapeutischen Entscheidungen bei der Behandlung von Spendern und Empfängern führen, wodurch die Zahl und die Sicherheit der Transplantationen erhöht werden.

**Table 1:** 

	Erfolgreiche Evaluation der Testproben	Zeit bis HE-Schnitt	Upload-Zeit	Remote-Befundung
Konventioneller Schnellschnitt	Ja (20/20)	Ca. 15 min	Ca. 5 min	Ja
FCM	Ja (20/20)	< 7 min	Ca. 1 min	Ja

Die Ersparnisse im Bereich Zeit/Personal vergleichend zwischen konventionellen Schnellschnitt vs. FCM erfolgte anhand von 20 Leberproben. Aktuell wird der konventionelle Schnellschnitt durch eine/einen medizinisch-technische(n) Assistenten/in im 24/7-Bereitschaftsdienst angefertigt, digitalisiert und per Upload dem(r) Pathologen(in) zur Remote-Befundung bereitgestellt

*FCM* fluoreszenzbasierte Konfokalmikroskopie, *HE* Hämatoxylin-Eosin

**Table 2:** 

	Probentransport notwendig (Zeitersparnis)	MTA 24/7 notwendig	Remote-Befundung	Vollständige Digitalisierung
Herkömmlicher Ablauf	Ja (–)	Ja	Nein	Nein
Status quo	Ja (–)	Ja	Ja	Nein
Ausblick: FCM – on site im OP	Nein (mehrere Stunden)	Nein	Ja	Ja

Der Probentransport bezeichnet den Transport der Probe vom externen Krankenhaus in das pathologische Institut

**Figure 4:**
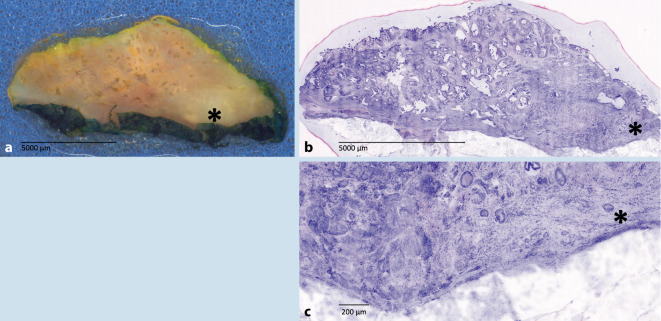

Die neu geschaffenen Möglichkeiten werden aktuell auch auf andere Fachbereiche ausgeweitet. So testen wir Anwendbarkeit der FCM bei HNO-Tumoren, in der Prostataschnellschnittdiagnostik sowie bei hämatoonkologischen Erkrankungen zur Beschleunigung der Lymphomdiagnostik. Erste präliminäre FCM-Scans sind in Abb. 4 und 5 dargestellt.

**Figure 5:**
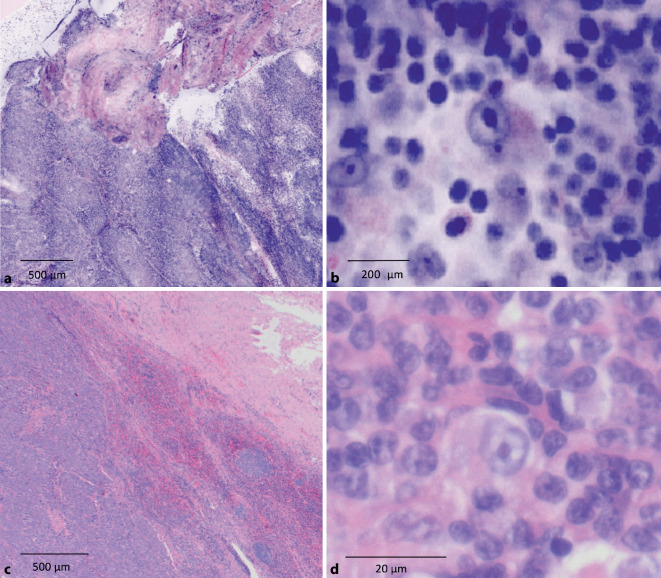

## Diskussion

Das SIP in Frankfurt arbeitet an einer vollständigen Digitalisierung der Abläufe. Die FCM-Technik als etablierte Methode und Alternative zum herkömmlichen Schnellschnittverfahren in der histopathologischen Evaluation von potenziellen Spenderlebern ist beispielhaft für die voranschreitende Digitalisierung der Pathologie. Insbesondere die Transplantationsmedizin durchläuft einen digitalen Wandel, und mit der Unterstützung einer digitalen Pathologie kann dieser Prozess erfolgreich voranschreiten. Während die Anschaffungskosten für ein FCM-Gerät bei etwa 350.000 € liegen, können Labore Material und Chemikalien der herkömmlichen Schnellschnittdiagnostik sowie oftmals fehlendes Personal im 24/7-Betrieb einsparen [17]. Außerdem können die Färbung und das Scannen von FCM-Proben nach einer Schulung auch von unerfahrenem Personal leicht durchgeführt werden. Dies ist insbesondere für den potenziellen Einsatz im OP-Saal wichtig, da das Färben und Scannen durch einen geschulten Mitarbeiter des DSO-Explantationsteams erfolgen könnte und somit den Weg zu einer virtuellen Schnellschnittdiagnostik in perioperativer Echtzeit ebnen würde. Bei entsprechender Infrastruktur können Gewebeproben vor Transplantation somit Spezialisten weltweit zur Online-Begutachtung bereitgestellt werden. Durch den perioperativen Einsatz würde der Probentransport ins pathologische Institut sowie das Vorhalten eines 24-h-MTA-Bereitschaftsdienstes wegfallen. Künstliche Intelligenz könnte in Zukunft ebenfalls eine wichtige Rolle bei der Bewertung von Transplantatproben spielen, um objektive, reproduzierbare und quantitative Daten zu liefern und die Früherkennung pathologischer Prozesse zu unterstützen [18]. Die Verknüpfung von digitalen HE-Schnitten, produziert mittels FCM in Echtzeit, und objektive Entscheidungsfindung mittels künstlicher Intelligenz, könnte die Grundlage für eine objektive und systematische Entscheidungshilfe in der pathologischen Routinediagnostik und insbesondere in der Transplantationspathologie bilden. Maschinelles Lernen könnte somit die Produktivität von Pathologen durch klinische Entscheidungshilfen und durchsetzungsfähige Arbeitsabläufe weiter steigern. FCM kombiniert mit maschinellem Lernen könnte somit einen Wendepunkt hin zur digitalen Transplantationspathologie darstellen, wodurch wichtige Endpunkte wie der frühe Transplantationserfolg und das Transplantatüberleben im Laufe der Zeit verbessert werden könnten.

## Fazit für die Praxis

- Die neuartige FCM-Technik (fluoreszenzbasierte Konfokalmikroskopie) erlaubt die Erstellung virtueller Hämatoxylin-Eosin(HE)-Schnitte von nativem Frischgewebe in Echtzeit.
- Die virtuellen HE-Schnitte können per Upload in ein passwortgeschütztes Portal von Pathologen weltweit begutachtet werden.
- Eine nachgeschaltete immunhistochemische oder molekularpathologische Diagnostik ist vollumfänglich möglich.
- Einem (aktuell) flächendeckenden Einsatz stehen die hohen Investitionskosten entgegen. Die laufenden Kosten (Färbematerial, Füllmaterial) der FCM sind jedoch als nachrangig einzustufen.
- Der perioperative Einsatz der FCM in Echtzeit im OP-Saal würde große Ersparnisse in Zeit und Personal ermöglichen.
- Die FCM könnte als Alternative zur herkömmlichen Schnellschnittdiagnostik als Grundpfeiler einer vollständigen Digitalisierung pathologischer Institute dienen.
